# Electro-optic Modulation in Polycrystalline Barium Titanate Metasurfaces Enhanced by Poling

**DOI:** 10.1021/acsphotonics.6c00340

**Published:** 2026-05-05

**Authors:** Eleni Prountzou, Helena C. Weigand, Virginia Falcone, Ülle-Linda Talts, Morgan Trassin, Rachel Grange

**Affiliations:** † ETH Zurich, Department of Physics, 27219 Institute for Quantum Electronics, Optical Nanomaterial Group, Zurich 8093, Switzerland; ‡ ETH Zurich, Department of Materials, Multifunctional Ferroic Materials, Zurich 8093, Switzerland

**Keywords:** electro-optic modulation, barium titanate, nanoimprint, metasurface, poling, tunable

## Abstract

Electrically tunable metasurfaces leveraging the strong Pockels effect in barium titanate (BaTiO_3_ or BTO) are a promising platform for reconfigurable free-space optical devices. However, the high cost, limited scalability, and restricted substrate compatibility of epitaxial BTO films hinder its exploitation. Here, we demonstrate free-space optical modulators based on imprinted BTO metasurfaces with targeted designs for optical and electric field confinement within the active material. With resonances exhibiting high quality factors of up to 200, we demonstrate improved transmission modulation at sub-volt driving amplitudes and frequencies up to 5 MHz. Additional enhancement is achieved via ferroelectric domain alignment, resulting in up to 25% higher modulation strength compared to the unbiased case and up to 75% compared to previous demonstrations. This enhanced EO response, arising from the effective permittivity engineering and domain orientation in these polycrystalline metasurfaces, holds significant potential for scalable and efficient EO modulators and active metasurfaces.

## Graphical abstract

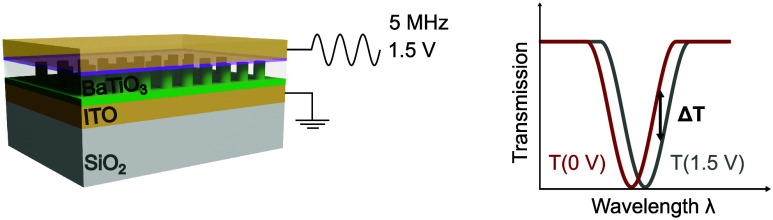

## 1 Introduction

Ferroelectric materials have recently attracted considerable interest due to their wide range of applications, including nonvolatile memories,^1,2^ ferroelectric photovoltaics,^3^ and acousto-optic or electro-optic (EO) devices.^4,5^ In telecommunications, where high-speed data transmission is essential, noncentrosymmetric materials with large EO coefficients and modulation bandwidths exceeding the kHz regime are required. While lithium niobate (LiNbO_3_) is widely used in both integrated^6^ and free-space devices, reaching modulation speeds above GHz,^7–9^ barium titanate (BaTiO_3_ or BTO) has gained attention due to its striking EO coefficient of *r*_42_ = 1300 pm/V (unclamped case) in bulk form.^10^ However, the limited integrability and scalability of bulk BTO have driven research toward attaining BTO films via techniques such as molecular beam epitaxy, metal–organic chemical vapor deposition, radio frequency sputtering, pulsed laser deposition, and spalling.^11–16^ Although epitaxial BTO on Si has achieved high-speed EO modulation with an EO coefficient of *r*_42_ = 923 pm/V,^17^ the epitaxial growth of layers remains energy-inefficient and restricted by substrate compatibility.

Conversely, wet-chemistry techniques, such as the sol–gel method, offer a versatile and scalable approach that is compatible with soft nanoimprint lithography (SNIL). This etch-free bottom-up process enables the imprinting of nanostructures on a variety of substrates and has been used for the fabrication of linear and nonlinear metasurfaces.^18–21^ Moreover, the polycrystallinity of these materials provides a simpler platform for designing and optically characterizing their effective polarization-independent EO performance.^22^ However, the randomly oriented grains of BTO lead to a lower effective EO coefficient of approximately 27 pm/V,^23^ which, nevertheless, remains comparable to that of monocrystalline LiNbO_3_.^24^

Further increases in the EO response of polycrystalline materials can be achieved through permanently orienting their ferroelectric domains, a process known as poling. At ambient temperature, BTO retains its tetragonal phase exhibiting a spontaneous polarization along the *c*-axis (out-of-plane) due to the displacement of the Ti^+^ ions along this axis within each unit cell.^10^ However, within a single crystal, multiple ferroelectric domains exist and can possess *a*-axis (in-plane) or *c*-axis orientation. In the BTO’s tetragonal phase, polarization reversal occurs via the formation of opposing 180° or orthogonal 90° domains. The high EO coefficient of monocrystalline BTO has prompted the investigation of domain effects within the perovskite oxide with recent achievements in domain alignment,^25,26^ as well as periodic poling^27^ of the crystal. Additionally, studies on ferroelectric switching in ultrathin BTO films show that both epitaxial and polycrystalline BTO thin films can achieve coercive fields and remanent polarization values approaching those of the bulk material.^28–30^ These advances are relevant for EO modulators, especially those based on polycrystalline BTO films and 3D structures, since a stable remanent polarization can introduce a built-in birefringence that enhances the EO response without requiring continuous biasing.

In this context, polarization stability has become an important figure of merit. While monocrystalline and epitaxial ferroelectrics such as BiFeO_3_ and PbTiO_3_ show retention periods of over a year,^31–33^ retention in polycrystalline films is mainly governed by grain boundaries, local electric field inhomogeneity, and grain-size-dependent domain stability.^34–36^ These are factors that contrast with the polarization relaxation driven by domain wall defects observed in single crystals. Solution-derived BTO allows for the tuning of these factors as adjusting parameters, such as the final annealing temperature, can be used to optimize the grain size to prolong polarization retention.^37^ Despite the complex domain dynamics associated with polycrystallinity, BTO remains competitive for device integration due to its relatively high effective EO coefficient. To further enhance this Pockels response, several studies in both single-crystalline and solution-processed polycrystalline BTO films have developed integrated EO modulators on lattice-matched buffer layers.^38,39^ This growth provides directionality and minimizes stresses in the grown BTO film, rendering such platforms promising for the future poling of EO devices.

Along the same direction of developing polycrystalline BTO-based modulators, a previous work reported a promising performance of an EO metasurface, showing potential for large-scale and high-speed free-space devices.^20^ The metasurfaces consisted of BTO pillars embedded in a SiO_2_ spacer layer. In this case, a glass substrate was used, with sandwiched top and bottom indium tin oxide (ITO) serving as transparent electrodes, enabling the characterization of the device’s transmission modulation. By applying an alternating current (AC) voltage of 1.5 V at a driving frequency of 400 kHz, the measured amplitude modulation reached 0.04%. However, the electric field was stronger in the low permittivity SiO_2_ capping layer rather than in the BTO pillars where the Pockels effect occurs. This suggests that optimized designs are required to further maximize the electric and optical field overlaps within the nonlinear material.

Driven by the potential that polycrystalline BTO devices have showcased for nanophotonic devices, in this study, we focus on engineering the effective permittivity of a transparent free-space EO modulator based on solution-processed polycrystalline BTO metasurfaces fabricated via SNIL. By designing sandwich-type device architectures that support high electric fields inside the active material and operate at low driving voltages (≤1.5 V), we enable ferroelectric domain alignment within nanostructured BTO, leading to enhanced transmission modulation. Furthermore, we show that EO modulation provides an indirect and accessible probe of the poling process and its temporal stability. These results establish a promising direction toward scalable and complementary metal-oxide-semiconductor (CMOS) compatible polycrystalline ferroelectric nanophotonic devices with enhanced EO performance.

## 2 Results and Discussion

The transparent free-space EO modulators studied here feature sandwich-type electrode configurations (Figure 1a) that host resonant metasurfaces consisting of arrays of solution-processed BTO nanopillars (Figure 1b). The nanostructures are fabricated via the etchless SNIL process using an inverse polydimethylsiloxane (PDMS) mold on fused quartz substrates, chosen to ensure minimal transmission losses. The imprinted structures are subsequently annealed at 800 °C for 2 h (fabrication and geometrical details are in the Experimental Section). More specifically, we investigate two distinct device architectures aiming to increase the electric field inside the BTO pillars. When the insulating planarization SiO_2_ layer is deposited in a thickness larger than the metasurface pillar height, the low permittivity SiO_2_ film captures most of the applied voltage, thereby diminishing the field in the high permittivity BTO.^20^ To mitigate this effect, one approach is to reduce the SiO_2_ planarization layer to match the height of the pillars (Figure 1c,d), hereafter referred to as the embedded device. Another solution we propose is to entirely eliminate the SiO_2_ layer and instead conformally deposit the ITO electrode on top of the BTO nanostructures (Figure 1e,f), hereafter termed the conformal device. In both approaches, a 20 nm-thick insulating layer of AlO_x_ is included between the BTO nanostructures and the top electrode to ensure electrical isolation during field application. Notably, both solutions maintain a low operating voltage of 1.5 V, as well as a small footprint of 50 × 50 μm^2^, which renders them compatible with CMOS driver circuits.^40^

**Figure 1:**
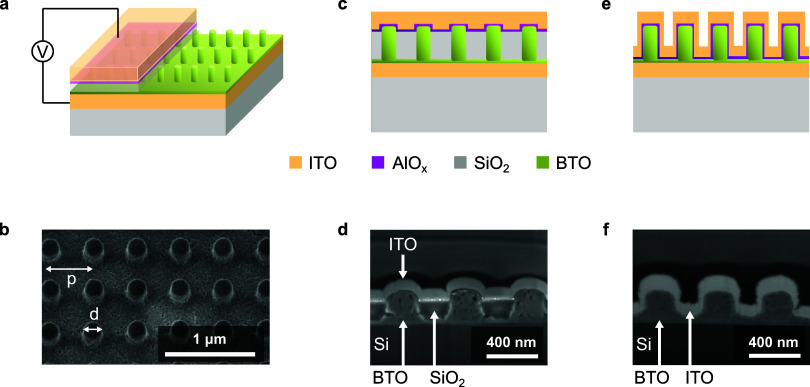
Structural configurations of barium titanate (BTO) metasurfaces. (a) Schematic of the device used to demonstrate electro-optical modulation of the transmitted signal under an applied AC voltage across the sandwiched ITO electrodes. The BTO pillars are buried in a SiO_2_ layer, and a conformal insulating AlO_x_ film is deposited on top of the capping SiO_2_ layer. (b) Scanning electron micrograph (SEM) (tilted at 30°) showing the top view of the imprinted metasurface consisting of arrays of BTO pillars with a periodicity of *p* = 500 nm and a diameter of *d* = 200 nm. (c) Sketch of the embedded configuration, where SiO_2_ is etched to the height of the pillars. (d) Cross-sectional SEM of the embedded device. (e) Schematic cross-section of the conformal configuration, where AlO_x_ and ITO are deposited conformally over the nanostructures. (f) Cross-sectional SEM of the conformal device. All of the cross-sectional SEM images were prepared using focused ion beam (FIB) milling. The devices shown in (d) and (f) are clone devices fabricated on a silicon substrate without a ground ITO electrode below the metasurface for better imaging quality.

To validate the effect of the device architecture on the EO performance, finite element method (FEM) simulations were performed for the two devices. The radius and periodicity of the meta-atoms were adjusted to position their resonances within the range of 750–790 nm, corresponding to the tunable laser used for the transmission modulation measurements. Due to differences in the device stacks, separate optimizations were required for each configuration (simulation details, including relative permittivity values, are in SI section 1). The electric and optical field distributions for the embedded and conformal devices at optimal wavelengths of 783.6 and 768.6 nm, respectively, are shown in Figure 2a–d. A positive potential of 1.5 V is applied on the top electrode for both devices to investigate the electric field distribution and estimate the refractive index change (Δ*n*) in BTO. However, it is noted that, as derived from the FEM simulations considering the relative permittivities of the materials (details in Section 1 of the SI), from the applied 1.5 V, approximately 0.6 V actually drops across the active material in the two devices, while the greater part of it falls on the AlO_x_ and SiO_2_ layers (Figure 2a,c). This indicates that although the conformal device exhibits a higher electric field within the BTO pillars, the larger pillar height in the embedded configuration compensates for this difference, leading to a comparable voltage drop across the BTO. Furthermore, these simulations treat the BTO residual film and nanopillars as a homogeneous medium. A more realistic representation, including randomly oriented domains, lower-permittivity grain boundaries, and air inclusions (as visible in the cross-sections in Figure 1d,f), is challenging to incorporate into the model. As a result, the estimated electric field in the active BTO material should be considered an upper boundary for this type of device, and the effective field in the ferroelectric material is likely to be lower. Overall, minimizing the proportion of these insulating layers is essential in order to increase the applied field in the metasurfaces.

**Figure 2:**
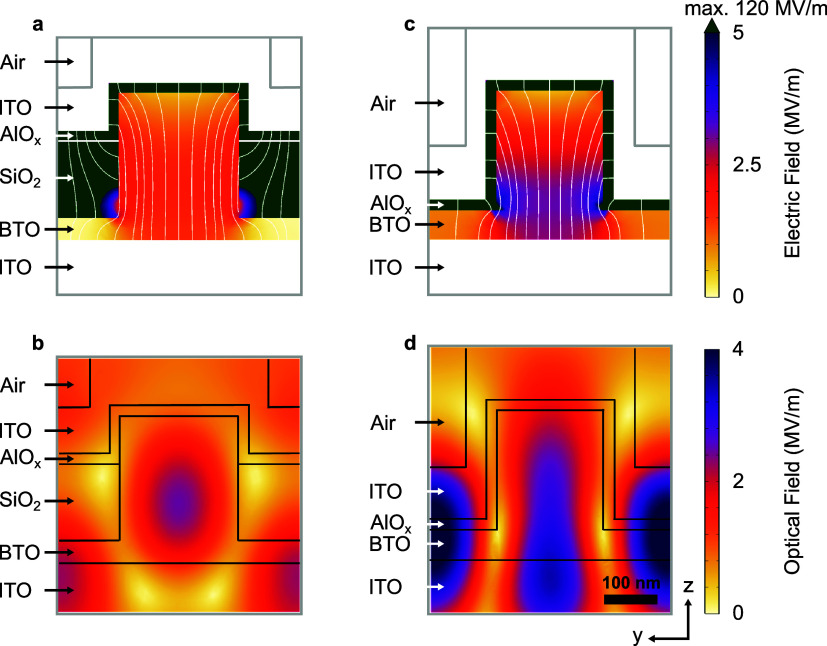
Electric and optical field simulations for the two electro-optic device configurations. Panels (a) and (b) show finite-element simulations of the electric and optical fields, respectively, for the embedded structure at 783.6 nm. Panels (c) and (d) correspond to the electric and optical field distributions, respectively, of the conformal structure at 768.6 nm. The electric field lines are overlaid in white in panels (a) and (c). The color scales for the electric field (a,c) and the optical field (b,d) are shown on the right for both device configurations. For the electric field, the color scale is saturated at 5 MV/m, with a maximum field strength of 120 MV/m in AlO_x_ and SiO_2_ (in green). The scale bar and coordinate axes shown at the bottom right of panel (d) are global and common for all simulations.

An optimal EO performance is supported by a spatial overlap that satisfies the electric and optical field distributions, which can be quantified by the overlap integral factor.^41^ The combination of the electric and optically induced fields exhibits a mode overlap integral of 27% for the embedded device and 37% for the conformal configuration. In addition, a strong electric field intensity in the BTO pillar indicates a corresponding increase in Δ*n*, considering the proportionality between Δ*n* and the applied electric field *E*, as given by the relation: $\Delta n=-\frac{1}{2}\cdot r_{\mathrm{eff}}\cdot n^{3}\cdot E$ , where *r*_eff_ is the effective EO coefficient and *n* is the effective refractive index of the material. In the case of the embedded nanostructures, the simulation (Figure 2a) predicts a 10-fold enhancement in the electric field intensity in the BTO pillar compared to the previous device,^20^ while for the conformal structure, the simulation shows a 13-fold enhancement (Figure 2c). Therefore, the simulated enhanced fields are expected to yield a comparable increase in Δ*n* and consequently in the modulation efficiency of the devices. Considering an average refractive index of 1.91 for our polycrystalline BTO in the wavelength range of 750–790 nm (as determined through ellipsometry measurements available in a previous study^20^), and an effective EO coefficient of 27 pm/V,^23^ the expected refractive index shift at the center of the BTO pillars is calculated to be approximately Δ*n*_embed_ = 1.8 × 10^–4^ for the embedded configuration (*E*_embed, BTO_ = 1.9 MV/m) and Δ*n*_conf_ = 2.3 × 10^–4^ for the conformal structure (*E*_conf, BTO_ = 2.4 MV/m).

To confirm the promising simulated results, the two metasurface-based free-space modulators were experimentally characterized by measuring the collinear transmission spectra and transmission amplitude modulation at normal incidence. A linearly polarized continuous-wave laser tunable in the range of 750–790 nm was utilized to probe the devices, and the signal was collected using a 50× objective and directed to both a camera (for imaging the sample) and a photodiode (for signal detection) via a 50/50 beam splitter. The photodiode output signal was guided to a lock-in amplifier, which simultaneously provided the driving AC frequency and amplitude for the electric field applied to the electrodes, while demodulating the transmitted signal (detailed in^20^).

Starting with the embedded configuration, a metasurface composed of 125 nm radius cylindrical pillars arranged in a square lattice with a 500 nm period was investigated. The experimentally measured resonance, shown in Figure 3a (gray line), exhibits a quality (Q) factor of 200 and a line width of 4 nm, as determined by fitting a Fano profile to the experimental data, which is in good agreement with the value of 207 calculated for the simulated resonance (fit details are in SI section 2). The Fano model was selected due to the asymmetric line shape, attributed to the coupling between lattice and Mie modes forming hybrid resonances.^20^ In both configurations, the data presented in Figures 3a,b were processed to suppress the Fabry–Pérot oscillations within the fused quartz substrate, which otherwise reduce the spectral clarity (details for the process are in SI section 3). In the same plot (Figure 3a), the EO modulation (red line) is shown as a function of the wavelength. The evaluation of the EO modulation efficiency is determined through the calculation of the relative transmission amplitude modulation as the ratio $\frac{\Delta T}{T}=\frac{T_{V=V_{\mathrm{pp}}}-T_{V=0}}{T_{V=0}}$ when a sinusoidal voltage waveform with a peak-to-peak amplitude of *V*_pp_ is applied at a given frequency. The EO efficiency reaches its maximum absolute value of 0.08% at the steepest transmission resonance slope, that is, when *dT*/*dλ* is largest.

**Figure 3:**
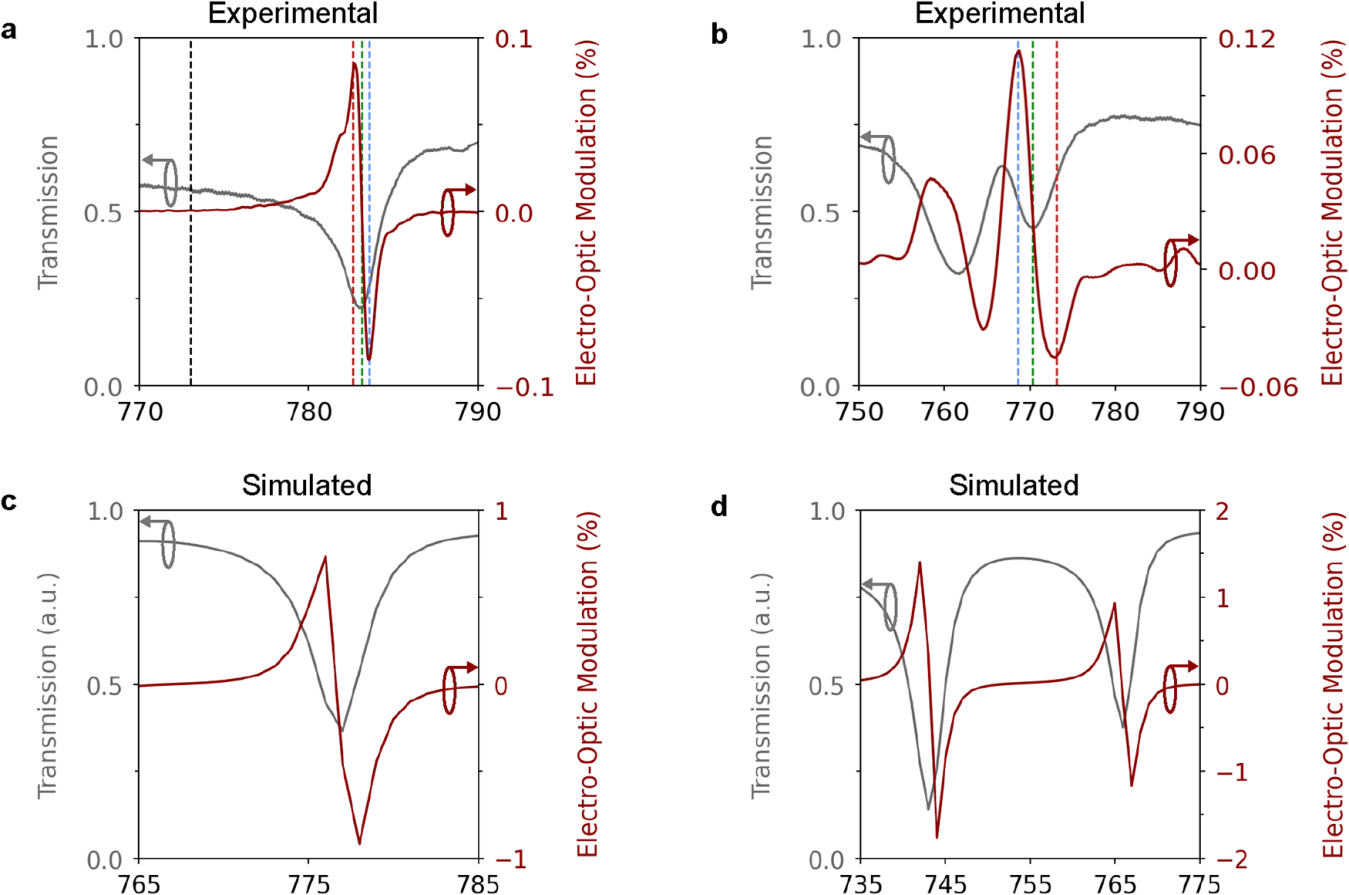
Experimental and simulated results for the embedded and conformal device configurations. Panels (a) and (b) illustrate the measured transmission spectrum (in gray) and electro-optic (EO) modulation (in red) of the embedded and conformal structures, respectively, when an electric field with an amplitude of 1.5 V and a driving frequency of 400 kHz is applied. The transmission spectra were obtained by sweeping the laser wavelength and acquiring the DC component of the demodulated signal (*T*_V_DC_ = 0_). The transmission data are normalized by the transmission of an unstructured region. The EO modulation is defined as the ratio $\frac{\Delta T}{T}=\frac{T_{V=V_{\mathrm{pp}}}-T_{V=0}}{T_{V=0}}$ , where *V*_pp_ is the peak-to-peak amplitude of the sinusoidal wave function applied at a specific frequency. It is noted that the sign of the modulation depends on the operating point along the resonance, as the field-induced refractive index change can either increase or decrease the transmission. Since this sign reflects the local slope of the spectrum rather than the intrinsic EO response, the absolute value of the modulation is reported when comparing device performance. The vertical dotted lines in the plots mark the different wavelengths at which the EO response was further examined in Figures S4a–d in the SI section 4. Panels (c) and (d) present the finite-element-simulated transmission spectra (in gray) and EO modulations (in red) of the embedded and conformal architectures, respectively.

For the conformal configuration to have a resonance in the near-infrared spectral range, the metasurface consisted of meta-atoms with 450 nm periodicity and 100 nm radius. Figure 3b shows the experimental EO modulation (red line) and the hybrid resonance (gray line), with a Q-factor of approximately 115 and a line width of 6.5 nm, which is on the same order of magnitude with the value of 204 calculated from the simulations (fit details are in SI section 2). In this design, the absence of the SiO_2_ layer increases scattering losses from the surface granularity of BTO, leading to a lower Q-factor. In contrast, embedding in glass strengthens the lattice resonant effects through phase-matched in-plane diffraction in the substrate and surrounding media and mitigates these roughness-induced losses.^42^ Nevertheless, the conformal device still achieves a stronger modulation of 0.12%, as the elimination of the low permittivity SiO_2_ layer enables a higher electric field inside the BTO nanopillars.

The experimental EO modulation values are compared against FEM simulations, which predict transmission modulations of 0.9 and 1.8% for the embedded (Figure 3c) and conformal (Figure 3d) designs, respectively. The deviations from the experimental results likely arise from fabrication-related imperfections (e.g., multiple scattering from surface roughness, porosity, and high conductivity). Despite the 20 nm-thick AlO_x_ coating, the insulation remains inadequate due to the inherent porosity of the solution-derived metal oxide, which is a result of the evaporation of organic subproducts during annealing, volume shrinkage, and incomplete grain coalescence.^43^ Strategies to reduce porosity include lowering the precursor concentration, which promotes a dense columnar microstructure,^44^ and performing multiple deposition and annealing cycles to progressively fill voids.^43^ In the context of our devices, this approach could be implemented by imprinting the BTO metasurfaces on top of a dense BTO thin film formed through multiple sol–gel deposition cycles.

Additionally, the reduced resistance of the devices, which was measured to be approximately on the order of 10 kΩ, far from the expected MΩ range that would ensure sufficient insulation, further contributes to the deviation between the experimental and simulated results. To reduce the conductivity of the devices and increase the effective electric field in the metasurfaces, the porosity of the BTO sol–gel must be targeted, as increasing the thickness of either of the low permittivity insulating layers (SiO_2_ or AlO_x_) would instead reduce the field strength inside the BTO pillars (Figure 2a,c).^20^ To improve the dielectric quality of the AlO_x_ layer, careful optimization of deposition temperature and postdeposition annealing (typically 300–400 °C in inert or forming gas atmospheres) could be implemented.^45,46^ Alternatively, replacing SiO_2_ with a higher relative permittivity oxide, such as TiO_2_, could reduce the electric field proportion in the insulator. However, it is imperative to emphasize that these results represent a substantial advancement compared to prior reported values for EO BTO metasurface modulators, with the embedded configuration achieving twice and the conformal design three times higher EO modulation.^20^

To further assess the devices’ performance, the dependence of the relative modulation on both voltage amplitude (at 400 kHz) (Figures S4a,b in SI section 4) and frequency (at 1.5 V) (Figure 4a) was examined for the two devices at the wavelengths corresponding to their maximum transmission modulation (additional data for all examined wavelengths are provided in SI section 4). In both cases, within the low voltage regime (≤1.5 V), the modulation scales linearly with the AC amplitude (Figures S4a,b in SI section 4), confirming the Pockels effect as the primary modulation mechanism. In addition, our devices yield up to 600 times greater modulation efficiency than their equivalent unpatterned films (data available in SI section 4). Complementary to the modulation amplitude response, our free-space EO modulators exhibit frequency bandwidths exceeding the kHz range, highlighting their potential for high-speed EO modulation (Figure 4a). It is evident that a strong modulation occurs at low frequencies, with a 3 dB drop near 1 MHz for both devices; however, for the embedded configuration, it remains stable up to 5 MHz. This frequency limitation originates not from the intrinsic EO effect but from the RC limitations of the device stack (electric circuit characterization in SI section 5). Improving the electrode’s conductivity or reducing the capacitor area could therefore extend the modulation bandwidth.^20,47^ In addition, further measurements across multiple metasurfaces and different device geometries are provided in section 6 of the SI, illustrating the repeatability of the observed EO response.

**Figure 4:**
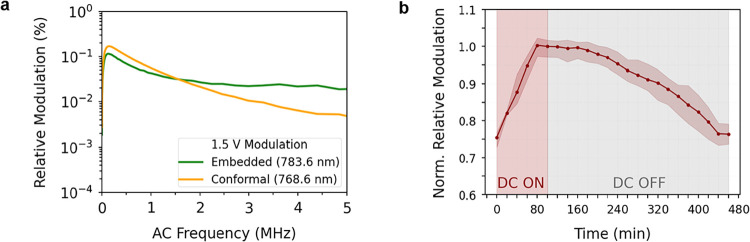
Experimental electro-optic performance of the embedded and conformal devices. (a) Relative modulation of both devices as a function of the applied AC driving frequency for a fixed amplitude of 1.5 V, measured at the optimal operating wavelengths of each device. (b) Temporal evolution of the normalized relative modulation of the conformal device under a DC bias of an absolute value of 10 V (33 MV/m) applied for 100 min, during which ferroelectric domain alignment enhances the electro-optic modulation. The values are normalized to the maximum modulation at the end of the poling step (*t* = 100 min, 0.16%) to emphasize stability. The markers denote the average over three independent poling cycles, while the shaded area indicates the spread between the minimum and maximum values. After the bias is removed, the modulation remains stable for one hour before gradually relaxing over a period of almost 5 h.

Beyond improving the device design for increased effective electric fields, permanent poling in polycrystalline ferroelectrics would allow a lasting modification of their birefringence, increasing the effective EO coefficient without the need for continuous DC biasing. The device architectures demonstrated here enable the alignment of ferroelectric domains in the nanostructured polycrystalline BTO, leading to enhanced EO performance and sustained stability. Figure 4b illustrates the evolution of the normalized relative modulation when a DC bias of an absolute value of 10 V (33 MV/m) is applied to the conformal device for 100 min, with the response recorded in intervals of 20 min by instantaneously turning off the DC voltage and recording the device’s AC response with a driving frequency of 400 kHz and an amplitude of 1.5 V (details for the embedded structure are in SI section 7). The modulation is normalized to its value at *t* = 100 min, corresponding to the maximum stable modulation amplitude (0.16%), to more clearly highlight the retention behavior. The reported data include statistics from three independent poling cycles on the same device, where the markers represent the mean value of the relative modulation, and the shaded region indicates the range between the minimum and maximum values. While a static electric field can drive the motion of both 90° and 180° domain walls, permanent polarization is generally associated with the switching of 180° domains, which requires sufficiently long poling durations to enable their motion.^48,49^ The reconfiguration of domains was observed to be saturated after 100 min of applying a static electric field, achieving 25% increase in modulation amplitude (initial value of 0.12%) (the collective results of the two devices are summarized in Table S2 of section 8 in the SI). This enhancement arises from the change in the birefringence of BTO, which results in a higher effective EO coefficient and therefore improves the relative modulation. As no additional enhancement in modulation amplitude was observed after 100 min, the DC bias was removed, and the domains were allowed to relax.

Following the poling process, the temporal stability of the device was monitored for several hours. During this period, the device maintained a stable modulation for about 1 h. After that, over a period of approximately 160 min, the modulation gradually declined to 90% of its maximum value, before eventually returning to its initial value. Such stability is challenging to achieve in this material due to the complex dynamics of its ferroelectric domains, which can backswitch upon removal of the bias, especially when the applied field is not sufficient to fully switch them.^50^ In polycrystalline ferroelectrics, backswitching arises from a combination of mechanisms, rather than a single dominant mechanism, including depolarization fields due to incomplete charge screening and ferroelectric–dielectric stacks,^51,52^ as well as domain-wall pinning and depinning at grain boundaries.^53^ Consequently, retention in these materials exhibits a broad distribution of relaxation times depending on the spatial variation of local switching conditions.^54,55^ In addition, secondary effects such as interfacial charge trapping at the BTO/AlO_x_ and BTO/SiO_2_ interfaces under high electric fields and electrochemical processes, such as oxygen vacancy migration and interfacial redox reactions, may locally modify internal fields and contribute to the relaxation dynamics.^56,57^ The use of oxide interlayers in the present structures is expected to mitigate direct chemical interactions between ITO and BTO, suggesting that the observed remanent modulation primarily originates from partial ferroelectric domain alignment, while nonferroelectric mechanisms may contribute to the relaxation dynamics. These results indicate that poling three-dimensional polycrystalline solution-derived BTO nanostructures is feasible and leads to improved EO performance with prolonged stability.

While long polarization retention has been extensively studied in monocrystalline epitaxial ferroelectric thin films,^31–33^ these investigations have mainly focused on planar geometries. In contrast, the direct investigation of poling and domain stability in nanostructured and metasurface-based polycrystalline materials remains experimentally challenging. In this work, EO modulation is employed as an indirect probe of the ferroelectric domain reconfiguration in BTO polycrystalline nanostructures, enabling the assessment of polarization stability in metasurfaces. Further increases could be achieved by improving the device resistance and electrode conductivity, which will allow the application of higher DC voltages and thereby promote stronger domain alignment. In addition, while at temperatures below the Curie point the applied field induces polarization switching through domain reorientation without any recrystallization, poling above the Curie temperature followed by cooling under bias can promote more stable domain configurations.^58–60^ Moreover, in monocrystalline epitaxial ferroelectric films, long polarization retention has been achieved through domain-wall pinning strategies, such as the introduction of nanoscale defects that apply a local compressive strain throughout the film, stabilizing the switched polarization.^31^ For epitaxial BTO, retention has been shown to depend on film thickness, with thinner layers (≈10 nm) exhibiting longer stability,^61^ while growing BTO on lattice-matched buffer layers was shown to improve the EO performance of integrated modulators.^38,39^ In this context, doping of BTO and the use of transparent lattice-matched substrates, such as MgO, or suitable buffer layers represent promising routes to improve stability in EO devices.

## 3 Conclusions

Resonant nanostructured BTO metasurfaces fabricated via the SNIL method have been shown to significantly enhance EO modulation compared to plain thin films, offering a scalable and etch-free route for optical devices.^20^ Although their modulation speed and electric field strength are superior to those of liquid crystals^62^ and phase change materials,^63^ their modulation amplitude was partly limited due to the electric field being stronger in the SiO_2_ planarization layer than in the BTO pillars.

In this work, to overcome this limitation, we develop two improved device configurations: an embedded design, where the SiO_2_ layer is etched up to the height of the pillars, and a conformal design, where the SiO_2_ capping layer is entirely omitted. These two configurations not only enable up to three times higher EO modulation compared to previous work,^20^ but also allow the poling of the BTO domains, resulting in a maximum increase of 25% of the relative modulation compared to the unbiased state, and a total improvement of 75% over a previously reported value.^20^ Moreover, the conformal device exhibits polarization stability for up to two hours, indicating the potential for sustained EO enhancement without the need for continuous DC biasing. Long-term retention reported in polycrystalline PZT, exceeding one year, further reinforces the potential of polycrystalline BTO for EO applications.^35^ Finally, by applying a driving voltage of only 1.5 V, we enable energy-efficient integration of these devices with CMOS-compatible technologies.

Furthermore, the oxide claddings used in our BTO metasurfaces (SiO_2_ and AlO_x_) are widely employed in integrated photonics across a variety of core materials, particularly with Si_3_N_4_, exhibiting comparable refractive indices to solution-derived BTO, supporting efficient optical confinement.^64,65^ In addition, a recent demonstration of monolithic solution-processed BTO waveguides using SNIL highlights a scalable route for integrating such materials into on-chip photonic circuits.^37^ This approach also enables ferroelectric domain poling within waveguides, supporting efficient electro-optic modulation. In this context, the incorporation of transparent conductive oxides, such as ITO, as conformal electrodes (as already demonstrated in integrated epsilon-near-zero modulators) further supports the feasibility of electrically driven integrated devices.^66^

Further optimization of the efficiency could be achieved via the implementation of high Q-factor resonances, such as quasi-bound states in the continuum, to maximize extinction ratios.^67,68^ For polycrystalline BTO, reducing the porosity would not only raise the material’s effective refractive index, thereby relaxing current design constraints for high-Q resonances, but would also improve the insulating properties of the nanostructures, enabling electric field distributions closer to those predicted by simulations. In this context, the SNIL method applied here offers a highly adaptable fabrication platform that is well-suited for a broad range of flat optical components. Recent demonstrations of metalenses based on solution-derived ferroelectrics^21,69^ highlight the potential of this approach, while the enhanced modulation efficiency achieved in this work provides a practical route toward developing high-speed, scalable EO metasurfaces and BTO-based metalenses, which have primarily been the subject of theoretical studies to date.^70–72^

## 4 Experimental Section

The complete process for the synthesis of the solution-derived BTO and its imprinting by SNIL, as well as the optical setup for the electro-optic characterization of the devices, can be found in previous work.^20^ Regarding the fabrication process, the cross-sections of both devices are illustrated in Figure 1c,e. The 100 nm-thick ITO electrodes were sputtered in a high vacuum on top of the 1 mm- and 500 μm-thick fused quartz substrates for the embedded and conformal devices, respectively. Subsequently, the BTO sol–gel was spin-coated for 5 s at 1000 rpm, and the metasurfaces were imprinted using a soft polydimethylsiloxane (PDMS) mold. After a 2 h curing of the samples at 70 °C, the molds were removed, and the samples were annealed at 800 °C for 2 h with a ramp rate of 2 °C/min to allow the BTO to transit from the amorphous to the polycrystalline phase. In the embedded configuration (Figure 1c), a 500 nm-thick spin-on glass was added as a capping layer to the BTO pillars and was heat-treated at 400 °C for 30 min. After that, reactive ion etching (RIE) of approximately 290 nm of excess SiO_2_ on top of the BTO meta-atoms was followed, until the protruding pillars appeared, as shown in Figure 1d. The RIE was performed at 20 mTorr by using a CHF_3_/Ar plasma gas. Finally, on both devices, a 20 nm AlO_x_ layer was deposited by atomic layer deposition, followed by the 100 nm-thick top electrode that was conformally sputtered on selected areas as the final step in the device fabrication. The selective deposition of the ITO top electrodes was achieved using photolithography to pattern the desired areas and a lift-off process.

The metasurfaces are composed of cylindrical pillar-shaped unit cells with engineered resonances across the visible and near-infrared spectra, which can be achieved by sweeping the periodicity (400–600 nm) and pillar diameters (100–300 nm). The pillars have heights of approximately 260 and 225 nm and residual layers of 45 and 55 nm for the embedded and conformal devices, respectively. As previously reported,^20^ the volumetric shrinkage of the imprinted nanostructures imposes a 0.5 filling factor limit to counteract this effect. The aspect ratios achieved here are close to 1.

## Supplementary Material



## Notes

Details of the simulations, Fano fitting of the resonances, Fabry–Pérot oscillations within the substrate cavity, AC response of the devices, electrical circuit characterization, electro-optic modulation across multiple metasurfaces and periodicity and radius sweeps, electro-optic response of the embedded device under DC biasing, and collective results of the performance of the two devices (PDF)
