# Supplementary material for: Electro-optic Modulation in Polycrystalline Barium Titanate Metasurfaces Enhanced by Poling
Source: ACS Photonics. 2026 May 5;13(10):2928–36. doi: 10.1021/acsphotonics.6c00340 (PMC13195732; doi:10.1021/acsphotonics.6c00340)
Supplement: Supplementary file 1 [file ph6c00340_si_001.pdf]

Supplementary Information

Electro-Optic Modulation in Polycrystalline Barium Titanate Metasurfaces Enhanced by Poling

Eleni Prountzou[1*], Helena C. Weigand[1], Virginia Falcone[1], Ülle-Linda Talts[1], Morgan Trassin[2], Rachel Grange[1]

[*] Corresponding Author: eprountzou@ethz.ch

[1] ETH Zurich, Department of Physics, Institute for Quantum Electronics, Optical Nanomaterial Group, 8093 Zurich, Switzerland

[2] ETH Zurich, Department of Materials, Multifunctional Ferroic Materials, 8093 Zurich, Switzerland

Contents

1. Simulations of electro-optic modulation S2

2. Fano fitting of resonances S3

3. Fabry-Pérot oscillations throughout the transmission spectra S4

4. AC voltage and frequency sweeps of the devices S6

5. Electrical circuit analysis S7

6. Electro-optic modulation across multiple metasurfaces and geometrical parameter sweep S12

7. Ferroelectric domains alignment of the embedded device S14

8. Collective results of the electro-optic response of the embedded and conformal devices S15

References S16

1. Simulations of electro-optic modulation

The electric field distribution of our devices, demonstrating the change in the refractive index (Δn), is shown in Figure 2(a) for the embedded configuration and in Figure 2(c) for the conformal configuration in the main text. From the finite element method (FEM) simulations, the electric field in the center of the BTO pillar is found to be approximately 1.9 MV/m for the embedded and 2.4 MV/m for the conformal structures when applying a driving voltage with an amplitude of 1.5 V. The relative permittivity values used in the simulations are as follows: $\varepsilon_{r,SiO_2} = 3.9$ for SiO_2, $\varepsilon_{r,AlO_x} = 6.7$ for AlO_x, and $\varepsilon_{r,BTO} = 500$ for BTO. For the latter, a lower value for the $\varepsilon_{r,BTO}$ was used compared to the values reported in literature ($\varepsilon_{r,BTO,lit.} \approx 1000$) [S1, S2] to account for the porosity and smaller ferroelectric domain sizes of our polycrystalline solution-derived nanostructures.

All FEM simulations were performed using geometries closely matching the fabricated device stacks (see Experimental Section of the main text). More specifically, for the embedded device, a period of 500 nm was used, with BTO nanopillars of 260 nm height and 125 nm radius, and a BTO residual layer thickness of 45 nm. The SiO_2 layer had a thickness of 160 nm. For the conformal device, the period used was 450 nm, with BTO pillars of 225 nm height and 100 nm radius, and a 55 nm-thick BTO residual layer. In both configurations, the AlO_x layer had a vertical and lateral thickness of 20 nm, while the ITO electrode thickness was 100 nm when planar and 40 nm laterally when deposited conformally on top of the pillars.

To quantify the spatial overlap of the electric and optical field distributions within the two devices (see Figures 2(a)-(d) of the main text), the electro-optic overlap integral factor, Γ, was computed using the following expression [S3]:

$$\Gamma = \frac{G}{V}\frac{\iiint |E_{op}(r)|^2 E_z \, dxdydz}{\iiint |E_{op}(r)|^2 \, dxdydz} = \Gamma_p + \Gamma_f = \frac{G_p}{V}\frac{\iiint |E_{op,p}(r)|^2 E_{z,p} \, dxdydz}{\iiint |E_{op,p}(r)|^2 \, dxdydz} + \frac{G_f}{V}\frac{\iiint |E_{op,f}(r)|^2 E_{z,f} \, dxdydz}{\iiint |E_{op,f}(r)|^2 \, dxdydz},$$

where G is the electrode gap, $V = 1.5\ V$ is the voltage applied on the electrodes, $E_{op}(r)$ is the optical field, and E_z is the electric field. Here, the integral range of the numerator includes only the electro-optic (EO) material (i.e., BTO), while that of the denominator includes the entire space between the electrodes. Since in both devices the electrodes distance around the pillar is not constant, the integral was calculated in two parts, as shown in the equation above: one around the pillar (denoted as Γ_p, with $G_{p,embed} = 325\ nm$ and $G_{p,conf} = 300\ nm$ being the electrode gap between the pillar for the embedded and conformal devices, respectively) and the other on the films surrounding the pillar (denoted as Γ_f, with $G_{f,embed} = 65\ nm$ and $G_{f,conf} = 75\ nm$ being the electrode gap between the films for the embedded and conformal devices, respectively).

2. Fano fitting of resonances

The experimental unprocessed data of the transmission dips of the embedded (Figure S2(a)) and conformal (Figure S2(b)) configurations, retrieved by sweeping the laser's wavelength, were fitted using a Fano profile [S4]. The fittings yield quality factors of 200 and 115, and linewidths of 4 nm and 6.5 nm for the embedded and conformal structures, respectively. The simulated transmission spectra of the embedded and conformal devices are presented in Figures S2(c) and S2(d), respectively. The parameters used for the FEM simulations are described in detail in Section 1. Fitting a Fano profile to those simulated data results in quality factors of 207 (with a linewidth of 3.7 nm) and 204 (with a linewidth of 3.8 nm) for the embedded and conformal configurations, respectively.

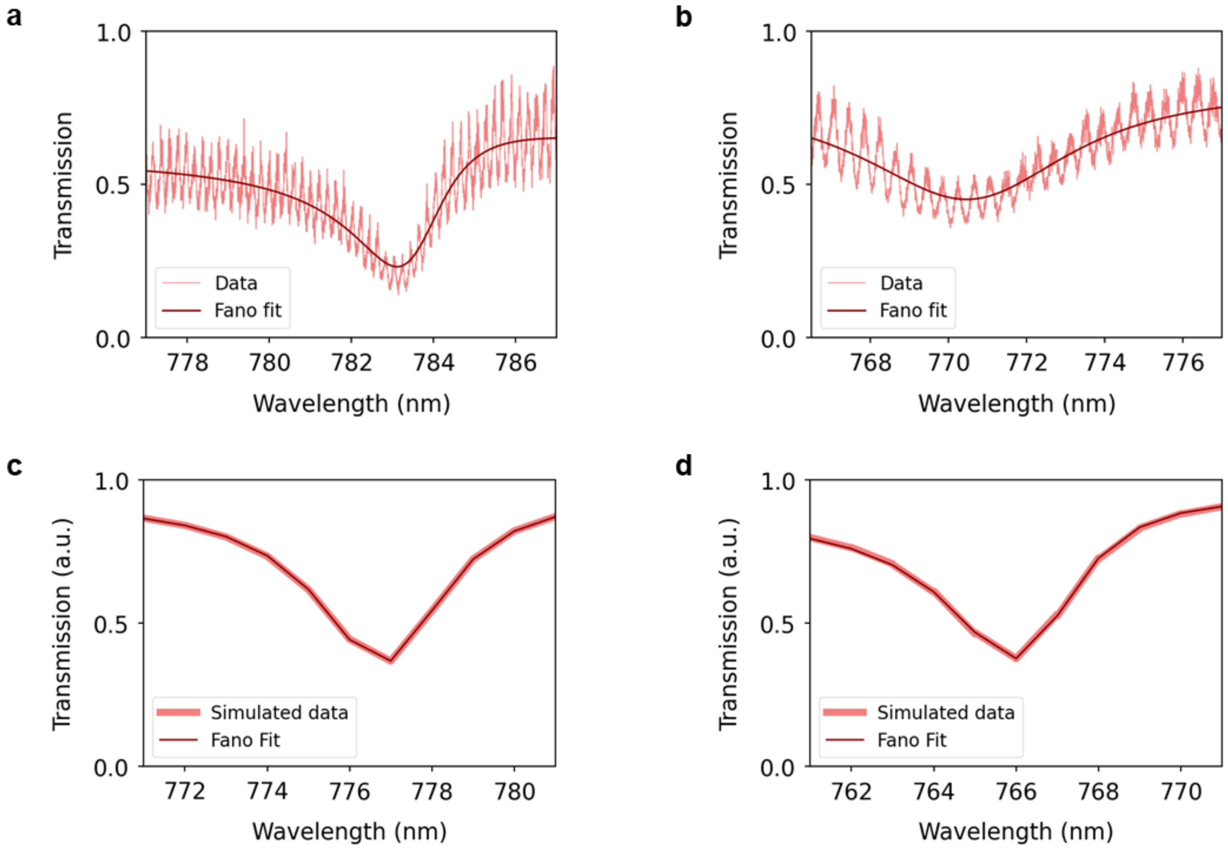

Figure S2: Fano profiles fitted on the experimental data for the (a) embedded and (b) conformal devices when an electric field with an amplitude of 1.5 V and a driving frequency of 400 kHz are applied, where the Fabry-Pérot oscillations are visible. All experimental transmission measurements are normalized with respect to an unstructured region of the sample. (c) and (d) show the Fano fits on the simulated transmission spectra for the embedded and conformal devices, respectively. In all cases, the transmission spectra for both devices are shown in light red and the corresponding Fano fits are plotted in dark red.

3. Fabry-Pérot oscillations throughout the transmission spectra

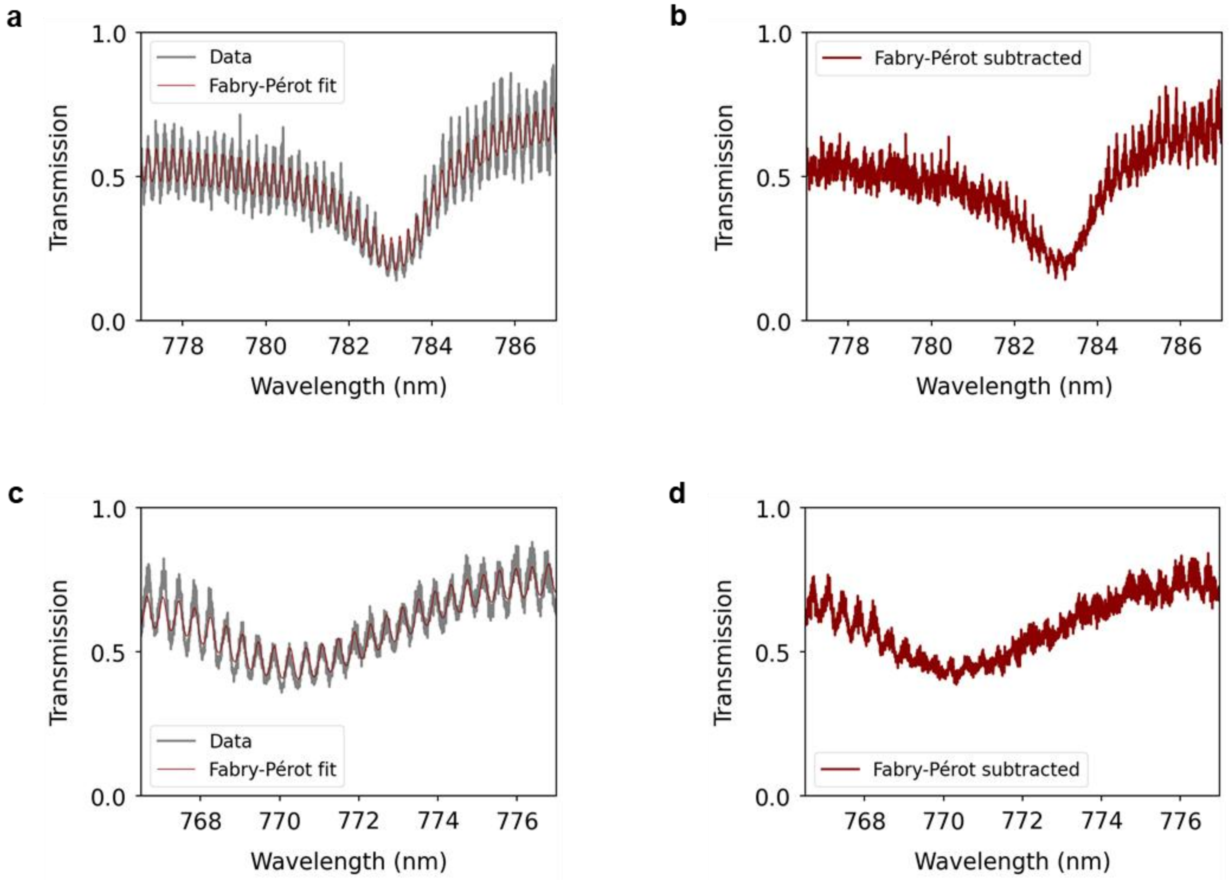

Figure S3: Fabry-Pérot oscillations in the transmission spectra (normalized to an unstructured region of the sample) of the embedded and conformal devices. The raw data (in gray) are fitted (in red) to extract the dominant free spectral range (FSR) for the (a) embedded and (c) conformal configurations. The obtained FSRs of 99.6 GHz for the embedded device and 204.5 GHz for the conformal device are consistent with the fused quartz substrate FSRs of 103 GHz and 207 GHz, respectively. Subtracting the fitted model from the raw data yields the residual spectra shown in (b) for the embedded device and (d) for the conformal configuration.

The experimental unprocessed data of the transmission spectra and the corresponding Fabry-Pérot (FP) fits are presented in Figures S3(a) and S3(c) for the embedded and conformal devices, respectively. For each device, the dominant free spectral range (FSR) associated with the FP oscillations was acquired. In the embedded configuration, the retrieved FSR is 99.6 GHz, while in the conformal device it is 204.5 GHz, both in good

agreement with the calculated values of 103 GHz and 207 GHz for the fused quartz substrate. The expected values are given by $FSR = \frac{c}{2nl}$, where c is the velocity of the light in vacuum, n is the refractive index of the optical cavity ($n_{SiO_2} = 1.45$), and l is the cavity's thickness ($l_{SiO_{2,embed}} = 1\ mm$, $l_{SiO_{2,conf}} = 500\ \mu m$).

Using the extracted FSRs, the FP oscillations were subtracted from the raw spectra, as shown in Figures S3(b) and S3(d) for the embedded and conformal devices, respectively. In both cases, a baseline obtained from filtering the raw data using a Savitsky-Golay polynomial function (see Figures 3(a), 3(b) of main text) was used to ensure the undisturbed fitting of the oscillations. However, because the amplitude of the FP oscillations varies across the resonance dip, the subtraction is not sufficient to fully remove the oscillatory features. For this reason, the filtered spectra are presented in the main text for improved clarity.

4. AC voltage and frequency sweeps of the devices

The AC voltage sweep at 400 kHz, as well as the AC frequency sweep at 1.5 V for all the examined wavelengths shown in Figures 3(a) and 3(b) of the main text are provided in Figures S4(a)-(d) for the embedded (Figures S4(a), S4(c)) and conformal (Figures S4(b), S4(d)) devices. In both configurations, the modulation exhibits a linear dependence on the applied AC voltage within the low voltage regime ($\leq 1.5\,V$) (Figures S4(a), S4(b)), confirming the Pockel's effect as the dominant modulation mechanism and proving that thermal or other type of drift effects are negligible within this range.

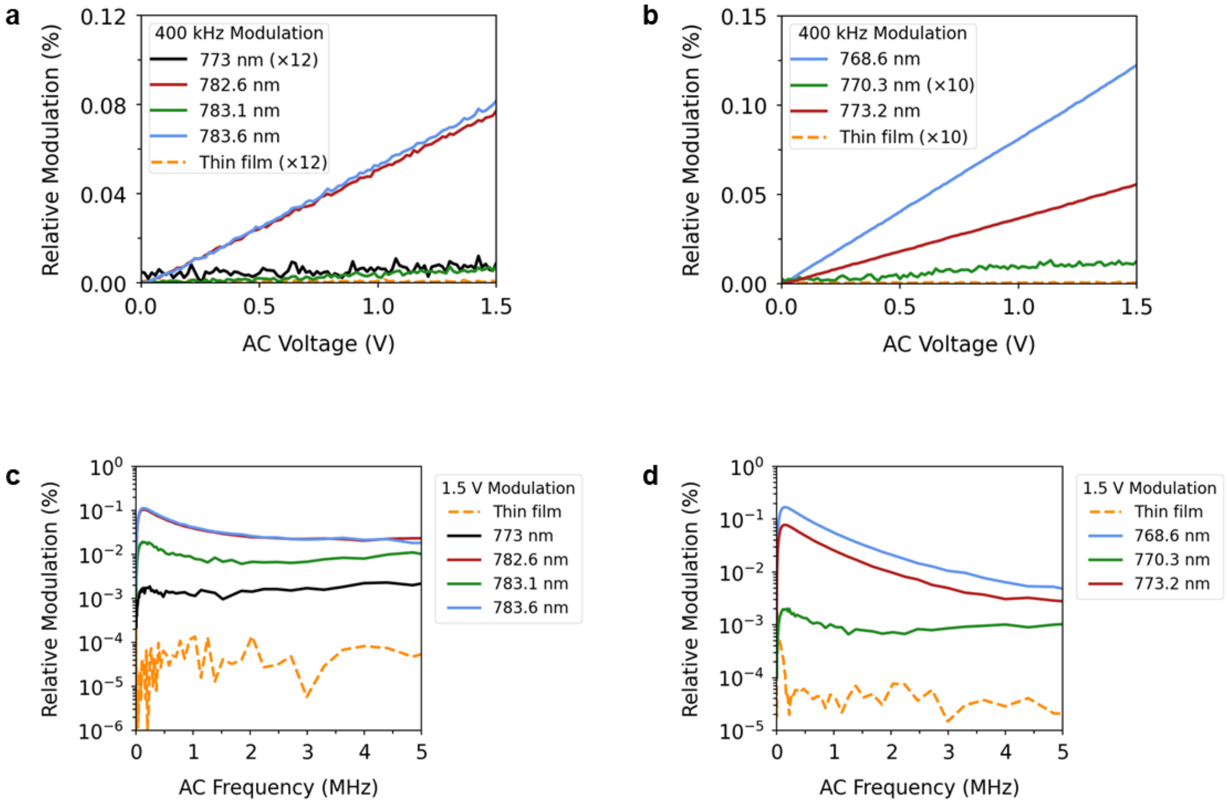

Figure S4: Experimental transmission modulation of the embedded and conformal devices. Panels (a) and (b) present the relative modulation at 400 kHz as a function of the applied voltage for the embedded and conformal structures, respectively. Note the scaling factors of 12 and 10 applied to the reference thin film and to certain low-signal wavelengths for the embedded and conformal devices respectively. Panels (c) and (d) show the relative modulation of the embedded and conformal devices, respectively, as a function of the applied AC driving frequency for a fixed amplitude of 1.5 V. In all plots, different wavelengths are represented by distinct colors, corresponding to the dotted lines of matching color in Figures 3(a) and 3(b) of the main text.

5. Electrical circuit analysis

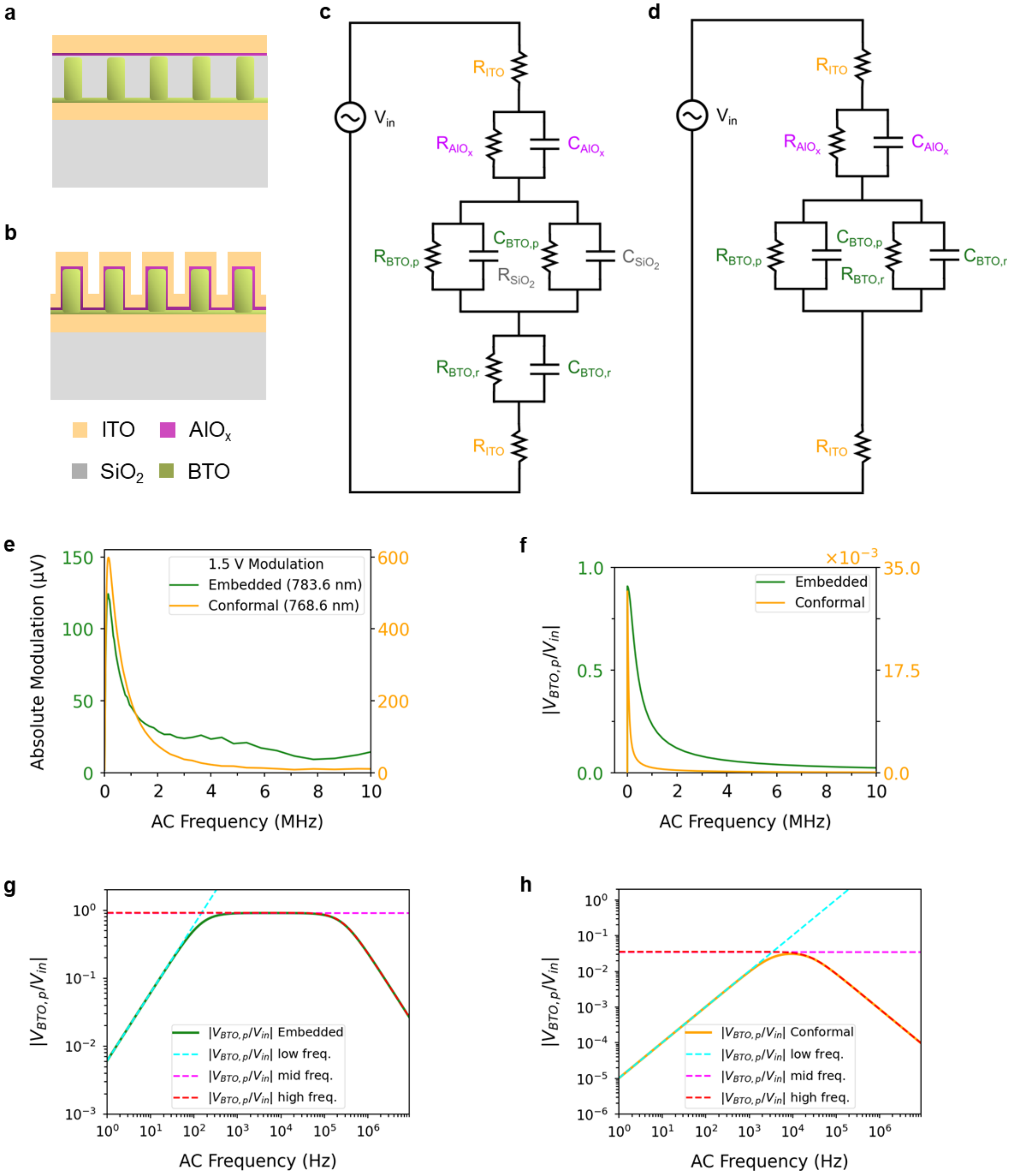

Figure S5: Electrical analysis of the stack of the embedded and conformal devices. Panels (a) and (b) show the cross-sectional schematics of the embedded and conformal devices, respectively. A simplified geometry was considered for the embedded device compared to

the fabricated structure (see the schematic in Figure 1(c) of the main text) to facilitate the electrical circuit computations. Panels (c) and (d) present the corresponding electrical circuits used for modelling the embedded and conformal devices, respectively. (e) Experimentally obtained optical modulation as a function of the AC frequency for the two devices at their respective optimal wavelengths. The modulation peaks occur at 136 kHz for the embedded structure and at 150 kHz for the conformal structure. (f) Linear Bode plot of the voltage drop across the BTO pillars, exhibiting a peak at low frequencies with maxima at 5 kHz and 9 kHz for the embedded and conformal devices, respectively. The presence of a corresponding low frequency peak in the optical response shown in (e) confirms that this feature originates from the electrical behavior of the devices. Panels (g) and (h) present the logarithmic Bode plots of the embedded and conformal devices, respectively, with the dotted lines indicating different frequency regimes of the equivalent circuit.

For examining the electrical behavior of the embedded and conformal devices in the different frequency regimes, the equivalent electrical circuits are presented in Figures S5(c) and S5(d), respectively. Schematics of the stack of the two devices are shown in Figures S5(a) and S5(b) for the embedded and conformal configurations, respectively.

Similarly to the analysis shown in literature [S5], each material is modeled individually from the rest as a resistor and capacitor in parallel. Considering the electric field lines normal to the electrodes for both cases, for the embedded device the BTO pillars and the capping SiO_2 layer can be handled as two different components with impedances $Z_{BTO,p}$ and Z_{SiO_2} in parallel as shown in Figure S5(c), while for the conformal device the same applies for the BTO pillars and BTO residual layer impedances that appear in parallel in Figure S5(d).

The resistance values for each material were computed by the formula

$$R_x = \rho_x \frac{t_x}{A_x},$$

where ρ_x is the resistivity, t_x is the thickness and A_x is the area of the xth layer of the device. An exception is the ITO electrodes for which the resistance was measured to be $R_{ITO} \approx 1\ k\Omega$. The capacitance of each layer was obtained using the formula

$$C_x = \frac{A_x \varepsilon_{r,x} \varepsilon_0}{d_x},$$

where A_x is the area, $\varepsilon_{r,x}$ is the relative permittivity, d_x is the thickness of the xth layer, and ε_0 is the dielectric permittivity of vacuum.

The total area covered by the electrodes is given by

$$A_{tot} = A_{SiO_2} + A_{BTO,p},$$

where A_{Si0_2} is the area of the embedding SiO_2 layer, which in the case of the conformal device is zero. $A_{BTO,p}$ is the total area covered by the pillars which is given by

$$A_{BTO,p} = N_{MS} \times N_{BTO,p} \times A_{sp},$$

where N_{MS} is the number of metasurfaces having ITO electrodes deposited on them, $A_{sp} = \pi R^2$ is the area of a single pillar with radius R, and $N_{BTO,p}$ is the number of pillars for each metasurface which is given by

$$N_{BTO,p} = \left(\frac{L_{MS}}{p}\right)^2,$$

where L_{MS} is the length of a single metasurface and p the periodicity of the pillars. For the embedded device the following values were used: $N_{MS,embed} = 20$, $R_{embed} = 125\ nm$, $L_{MS,embed} = 50\ \mu m$, $p_{embed} = 500\ nm$, while for the conformal device the values used are: $N_{MS,conf} = 18$, $R_{conf} = 100\ nm$, $L_{MS,conf} = 50\ \mu m$, $p_{conf} = 450\ nm$. The computed values for the area and the resulting resistance and capacitance for each layer are presented in Table S1.

Considering the electrical circuits in Figures S5(c) and S5(d), the voltage drop over the pillars for the two devices is given by the following expressions:

Embedded device: $V_{BTO,p,embed} = V_{in} \frac{Z_{tot,embed}}{2R_{ITO}+Z_{AlO_x}+Z_{tot,embed}+Z_{BTO,r}}$,

where $Z_{tot,embed} = \frac{Z_{SiO_2}\, Z_{BTO,p}}{Z_{SiO_2}+Z_{BTO,p}}$

Conformal device: $V_{BTO,p,conf} = V_{in} \frac{Z_{tot,conf}}{2R_{ITO}+Z_{AlO_x}+Z_{tot,conf}}$,

where $Z_{tot,conf} = \frac{Z_{BTO,p}\, Z_{BTO,r}}{Z_{BTO,p}+Z_{BTO,r}}$.

To define the factors that limit the frequency response of our devices, we calculated their behavior using the Laplace transform with the impedance of the xth component given by

$$Z_x = \frac{R_x}{1+sC_xR_x}.$$

Figures S5(g), S5(h) and S5(f) present the simulated frequency-dependent Bode magnitude of the embedded and conformal devices, plotted on logarithmic and linear scales, respectively. These simulations offer qualitative interpretation of the low frequency peak observed in the optical modulation response of the devices (Figure S5(e)). The shift of the electrical peak to lower frequencies compared to the optical peak (Figures S5(e), S5(f)), as well as the shorter plateau present in the Bode plot (Figure S5(h)) of the conformal device,

arise from the simplified assumption that the electric field is oriented strictly perpendicular to the electrodes. As shown by the FEM simulations in Figures 2(a) and 2(c) of the main text, a substantial contribution to the electric field (especially in the case of the conformal device) originates from the side of each pillar, which is not captured in this simplified analytical model. Regardless of this assumption, the proposed electrical circuit analysis reveals the main factors limiting the frequency response of the devices.

In the low frequency regime, the response can be described by a simple high-pass filter:

Embedded device: $$\left.\frac{V_{BTO,p}}{V_{in}}\right|_{low\ freq.,embed} = s\ R_{BTO,p}\ C_{AlO_x}$$

Conformal device: $$\left.\frac{V_{BTO,p}}{V_{in}}\right|_{low\ freq.,conf} = s\ R_{BTO,r}\ C_{AlO_x}\ .$$

In both configurations, the large AlO_x capacitance contributes to this filtering. In the embedded device the voltage drop across the BTO pillars is additionally dominated by the resistance of the pillars themselves, whereas in the conformal structure the dominant contribution arises from the resistance of the BTO residual layer. As a result, most of the applied voltage in the conformal device drops across the residual layer rather than the BTO pillars, which explains the significantly lower voltage drop over the pillars observed in both the linear and logarithmic Bode plots (Figures S5(f)-(h)) compared to the embedded device.

In the mid frequency regime, when the impedance of a capacitive element becomes smaller than its resistive counterpart, the circuit response is dominated by capacitances. For the embedded device, the effective capacitance is given by the parallel combination of the SiO_2 layer and BTO pillars, which is in series with the AlO_x capacitance. The conformal device is described by a simple capacitive divider between the AlO_x layer and the BTO residual layer. Accordingly, the voltage drop across the BTO pillars can be expressed as:

Embedded device: $$\left.\frac{V_{BTO,p}}{V_{in}}\right|_{mid\ freq.,embed} = \frac{C_{AlO_x}}{C_{AlO_x}+C_{SiO_2}+C_{BTO,p}}$$

Conformal device: $$\left.\frac{V_{BTO,p}}{V_{in}}\right|_{mid\ freq.,conf} = \frac{C_{AlO_x}}{C_{AlO_x}+C_{BTO,r}}\ .$$

Therefore, reducing the thickness of the insulating layers (i.e., AlO_x and SiO_2) or employing cladding materials with higher relative permittivity would increase the electric field inside the BTO pillars. Interestingly, in the logarithmic Bode plot (Figure S5(h)), the conformal device exhibits a significantly narrower mid-frequency plateau compared to the broad plateau observed for the embedded device (Figure S5(g)). This indicates that, in the conformal configuration, the frequency response is mainly governed by the low and high frequency regimes, whereas the mid frequency contribution is much less pronounced.

Lastly, in the high frequency regime, the response can be described as the contribution of the effective capacitance term derived in the mid frequency range, and a low-pass filter term:

Embedded device: $$\left.\frac{V_{BTO,p}}{V_{in}}\right|_{high\ freq.,embed} = \frac{C_{AlO_x}}{C_{AlO_x}+C_{SiO_2}+C_{BTO,p}}\ \frac{1}{1+s\ 2R_{ITO}\left(\frac{C_{AlO_x}\left(C_{SiO_2}+C_{BTO,p}\right)}{C_{AlO_x}+C_{SiO_2}+C_{BTO,p}}\right)}$$

Conformal device: $$\left.\frac{V_{BTO,p}}{V_{in}}\right|_{high\ freq.,conf} = \frac{C_{AlO_x}}{C_{AlO_x}+C_{BTO,r}}\ \frac{1}{1+s\ 2R_{ITO}\left(\frac{C_{AlO_x}\,C_{BTO,r}}{C_{AlO_x}+C_{BTO,r}}\right)}\ .$$

As in the mid frequency regime, the high frequency response of the two devices is limited by the AlO_x and SiO_2 capacitances. In addition, the resistance of the ITO electrodes becomes non-negligible and further limits their frequency response. Consequently, reducing the resistance of the electrodes or the thickness of the insulating layers would enable more efficient operation of the two devices at high frequencies.

Material	A [mm^2]	t_x [nm]	ρ_x [Ωm]	ε_r	R_x [Ω]	C_x [F]
**Embedded Device**						
BTO,r (residual)	1.2	45	10^4 [S6]	500	364	121.5×10^{-9}
AlO_x	1.2	20	10^{14} [S7]	6.7	1.6×10^{12}	3.7×10^{-9}
SiO_2	1.2	260	10^{15} [S8]	3.9	2.1×10^{14}	162.7×10^{-12}
BTO,p (pillar)	0.01	260	10^4 [S6]	500	2.7×10^5	167.2×10^{-12}
**Conformal Device**						
BTO,r (residual)	1.1	55	10^4 [S6]	500	490	90.4×10^{-9}
AlO_x	1.1	20	10^{14} [S7]	6.7	1.8×10^{12}	3.3×10^{-9}
BTO,p (pillar)	0.01	225	10^4 [S6]	500	3.2×10^5	137.4×10^{-12}

Table S1: Relevant parameter values, along with the resulting capacitances and resistances for each component in the modeled electrical circuits of the embedded and conformal devices.

6. Electro-optic modulation across multiple metasurfaces and geometrical parameter sweep

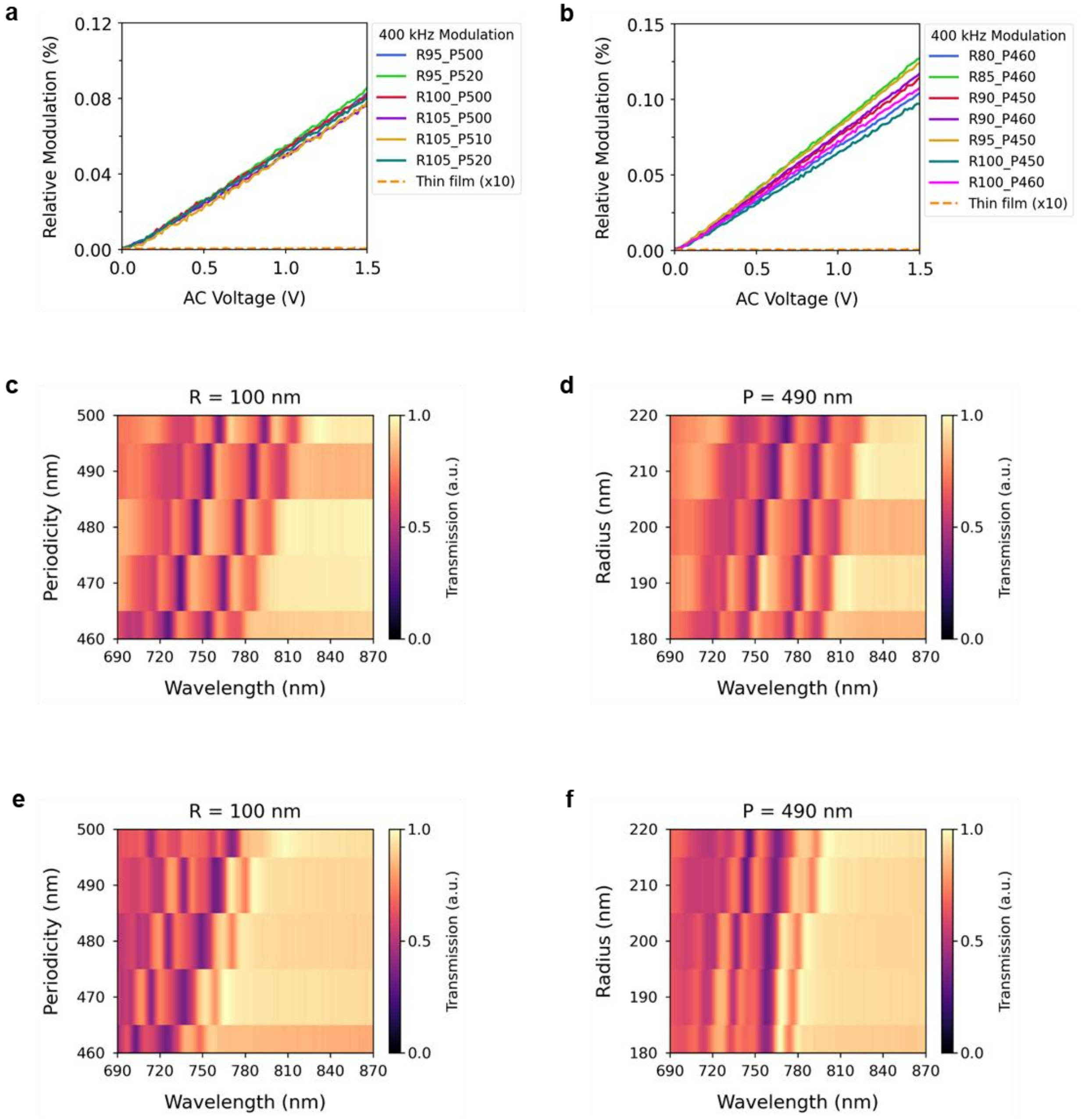

Figure S6: Electro-optic modulation and geometrical parameter sweeps of multiple metasurfaces. Relative modulation as a function of AC voltage (at 400 kHz) for different metasurfaces on the (a) embedded and (b) conformal devices. The notation in the legends follows the structure of RX_PY, where X is the pillar radius in nm and Y is the metasurface period in nm. The modulation remains on the same order of magnitude as the individual

cases discussed in the main text. For reference, a thin film of the same thickness is included in both plots, with its signal multiplied by ten for visibility. (c, e) 2D transmission spectra of two different samples with multiple metasurfaces of radius 100 nm, while the periodicity is varied between 460–500 nm. (d, f) 2D transmission spectra of the same samples for a fixed periodicity of 490 nm, while the radius is swept between 180–220 nm. All imprints were fabricated under similar conditions on quartz substrates with a 100 nm-thick ITO bottom electrode.

Figure S6 provides an overview of the EO performance and optical resonances across multiple metasurfaces on both embedded and conformal devices, as well as on different samples with similar fabrication conditions. Figures S6(a) and S6(b) show the relative modulation as a function of the applied AC voltage (at 400 kHz) for multiple metasurfaces on the embedded and conformal devices, respectively. These measurements confirm that the modulation remains on the same order of magnitude across different metasurfaces (≈0.08 % for the embedded device and ≈0.12 % for the conformal device), demonstrating reproducibility of the EO response. For comparison, the response of a thin film of equivalent thickness is also shown (signal multiplied by ten for visibility), highlighting the enhanced modulation provided by the nanostructured metasurfaces.

Figures S6(c) and S6(e) present the 2D transmission spectra of two separate samples, each containing multiple metasurfaces with a fixed radius of 100 nm while the periodicity is swept between 460-500 nm. These plots illustrate how small variations in periodicity influence the resonance positions and the spectral response, providing insight into the tunability of the hybrid Mie/lattice resonances. Figures S6(d) and S6(f) show the transmission spectra of the same two samples for a fixed periodicity of 490 nm while varying the radius between 180-220 nm, demonstrating the effect of radius on the resonance wavelength and spectral features. Overall, the comparison of the two similar samples in Figures S6(c)-(f) reveals minor differences in resonance positions and Q-factors (mean value of $\approx 88 \pm 29$), which can be attributed to the intrinsic variability of the manual imprinting process used in this study. It is noted that the use of wafer-scale nanoimprint tools is expected to significantly improve the process uniformity and consistency. Despite these slight differences, the general trends are consistent with the performance reported in the main text. Notably, the embedded device achieves a higher Q-factor (200), indicating that the surrounding glass cladding smooths the surface granularity of the polycrystalline BTO metasurfaces, whereas non-embedded structures exhibit lower Q-factors (≈100) due to enhanced roughness and scattering.

7. Ferroelectric domains alignment of the embedded device

The evolution of the normalized EO modulation when applying a static electric field to the embedded device is presented in Figure S7. Likewise to the conformal configuration, a DC voltage of an absolute value of 10 V (31 MV/m) was applied to the device for 100 minutes and its response was recorded every 20 minutes. The modulation is normalized to its maximum value at the end of the poling process ($t = 100\,min$, 0.09 %) to facilitate comparison of the retention behavior. After removal of the bias, the device maintains a nearly constant modulation for approximately one hour, followed by a gradual decrease towards its initial value (0.08 %) over a period of several hours (the device reaches 90 % of its maximum modulation value after a period of 220 minutes).

By aligning the ferroelectric domains in the polycrystalline BTO, an 11 % and 55 % enhancement in the EO modulation is observed compared to the randomly oriented domains in the unbiased case and the previously reported results with the non-optimized device, respectively [S5]. As mentioned in the main text, the presence of the low permittivity SiO_2 planarization layer prevents the performance of the embedded device to reach the response of its conformal counterpart.

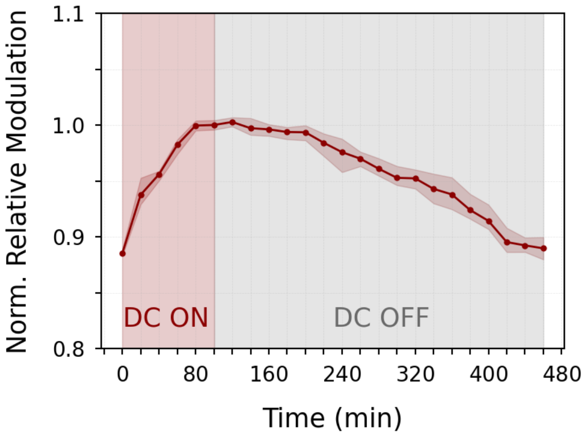

Figure S7: Time evolution of the normalized relative modulation of the embedded device during and after application of a DC bias of an absolute value of 10 V for 100 minutes. The modulation is normalized to its maximum value at $t = 100\,min$ (0.09 %). After removal of the bias, the modulation remains stable for approximately one hour before gradually relaxing towards its initial value ($\approx$0.08 %).

8. Collective results of the electro-optic response of the embedded and conformal devices

The collective experimental results for the embedded and conformal devices presented in this work, compared with the previously demonstrated results [S5], are summarized in Table S2. Notably, all devices comprise of a $50 \times 50\,\mu m^2$ metasurface, ensuring consistent comparison across different devices. The relative modulation is defined as $\Delta T/T = \frac{T_{V=V_{pp}} - T_{V=0}}{T_{V=0}}$, where V_{pp} is the peak-to-peak amplitude of the sinusoidal voltage applied at a specific frequency. The voltage of 1.5 V reported in the main text corresponds to the amplitude of the waveform, and therefore the peak-to-peak value is twice this amplitude. When operating at the steepest slope of the transmission resonance (where $dT/d\lambda$ is largest), the relative modulation reaches a maximum absolute value of 0.08 % for the embedded device and 0.12 % for the conformal device. This represents a twofold increase for the embedded device and a threefold increase for the conformal device compared to the previous work (0.04 %) [S5]. Upon poling the embedded device with a DC field of an absolute value of 10 V applied for 100 minutes, the maximum relative modulation increases to 0.09 %, corresponding to an 11 % improvement over the initial unbiased value (0.08 %) and a 55 % improvement compared to the previously reported value (0.04 %) [S5]. Similarly, poling the conformal device under the same conditions results in a maximum relative modulation of 0.16 %, which is 25 % higher than the initial unbiased value (0.12 %) and 75 % higher than the previously reported value (0.04 %) [S5]. After removing the DC bias, the enhanced relative modulation in both devices remains stable for approximately one hour, after which it gradually decreases over several hours until returning to its initial value.

Device Configuration	Voltage amplitude of AC field [V]	Frequency of AC field [kHz]	Maximum ΔT/T [%]	Maximum ΔT/T via poling [%]	Stability time of ΔT/T (%) after DC bias removal [hours]
Embedded (from Ref. [S5])	1.5	400	0.04	-	-
Optimized Embedded (this work)	1.5	400	0.08	0.09	≈ 1
Conformal (this work)	1.5	400	0.12	0.16	≈ 1

Table S2: Summary of the experimental electro-optic performance of embedded and conformal imprinted BTO metasurface devices. The table reports the applied AC voltage amplitude and frequency, the maximum relative modulation ΔT/T, the enhancement achieved via DC poling when applying a bias of an absolute value of 10 V, and the observed retention time of the relative modulation after removal of the DC bias.

References

(S1) Stawski, T. M.; Vijselaar, W. J.; Göbel, Ole F.; Veldhuis, S. A.; Smith, B. F.; Blank, D. H.; ten Elshof, J. E. Influence of high temperature processing of sol–gel derived barium titanate thin films deposited on platinum and strontium ruthenate coated silicon wafers. *Thin Solid Films* **2012**, *520*, 4394–4401.

(S2) Panomsuwan, G.; Manuspiya, H. Structural and dielectric properties of sol–gel derived $Ba_{1-x}Sr_xTiO_3$ ($0 \leq x \leq 0.5$) ceramics for energy storage applications. *Materials Research Express* **2018**, *6*, 026310.

(S3) Xiong, C.; Pernice, W. H. P.; Ngai, J. H.; Reiner, J. W.; Kumah, D.; Walker, F. J.; Ahn, C. H.; Tang, H. X. Active Silicon Integrated Nanophotonics: Ferroelectric $BaTiO_3$ Devices. *Nano Letters* **2014**, *14*, 1419–1425, PMID: 24447145.

(S4) Limonov, M. F. Fano resonance for applications. *Advances in Optics and Photonics* **2021**, *13*, 703–771.

(S5) Weigand, H. C.; Talts, Ü. L.; Vieli, A.-L.; Vogler-Neuling, V. V.; Nardi, A.; Grange, R. Nanoimprinting Solution-Derived Barium Titanate for Electro-Optic Metasurfaces. *Nano Letters* **2024**, *24*, 5536–5542, PMID: 38657957.

(S6) Edmondson, B. I.; Kwon, S.; Lam, C. H.; Ortmann, J. E.; Demkov, A. A.; Kim, M. J.; Ekerdt, J. G. Epitaxial, electro-optically active barium titanate thin films on silicon by chemical solution deposition. *Journal of the American Ceramic Society* **2020**, *103*, 1209–1218.

(S7) Groner, M.; Elam, J.; Fabreguette, F.; George, S. Electrical characterization of thin Al_2O_3 films grown by atomic layer deposition on silicon and various metal substrates. *Thin Solid Films* **2002**, *413*, 186–197.

(S8) El-Kareh, B. *Fundamentals of Semiconductor Processing Technology*; Springer New York, NY, **1995**.
